# Myocardial Overexpression of *Mecr*, a Gene of Mitochondrial FAS II Leads to Cardiac Dysfunction in Mouse

**DOI:** 10.1371/journal.pone.0005589

**Published:** 2009-05-18

**Authors:** Zhijun Chen, Hanna Leskinen, Erkki Liimatta, Raija T. Sormunen, Ilkka J. Miinalainen, Ilmo E. Hassinen, J. Kalervo Hiltunen

**Affiliations:** 1 Biocenter Oulu and Department of Biochemistry, University of Oulu, Oulu, Finland; 2 Biocenter Oulu and Department of Pharmacology and Toxicology, University of Oulu, Oulu, Finland; 3 Department of Medical Biochemistry and Molecular Biology, University of Oulu, Oulu, Finland; 4 Biocenter Oulu and Department of Pathology, University of Oulu, Oulu, Finland; Instituto de Química, Universidade de São Paulo, Brazil

## Abstract

It has been recently recognized that mammalian mitochondria contain most, if not all, of the components of fatty acid synthesis type II (FAS II). Among the components identified is 2-enoyl thioester reductase/mitochondrial enoyl-CoA reductase (Etr1/Mecr), which catalyzes the NADPH-dependent reduction of *trans-*2-enoyl thioesters, generating saturated acyl-groups. Although the FAS type II pathway is highly conserved, its physiological role in fatty acid synthesis, which apparently occurs simultaneously with breakdown of fatty acids in the same subcellular compartment in mammals, has remained an enigma. To study the *in vivo* function of the mitochondrial FAS in mammals, with special reference to *Mecr*, we generated mice overexpressing *Mecr* under control of the mouse metallothionein-1 promoter. These *Mecr* transgenic mice developed cardiac abnormalities as demonstrated by echocardiography *in vivo*, heart perfusion *ex vivo*, and electron microscopy *in situ*. Moreover, the *Mecr* transgenic mice showed decreased performance in endurance exercise testing. Our results showed a ventricular dilatation behind impaired heart function upon *Mecr* overexpression, concurrent with appearance of dysmorphic mitochondria. Furthermore, the data suggested that inappropriate expression of genes of FAS II can result in the development of hereditary cardiomyopathy.

## Introduction

Progress in molecular cardiology has revealed circumstances predisposing individuals to development of cardiomyopathies. Many of the genetic factors identified involve receptors in the myocardial sarcolemma, ion channels, and the cytoskeleton or contractive machinery. Energy metabolism restrictions, arising from mitochondriopathies or other inborn errors, frequently show a cardiac phenotype.

In mammalian cells, a vast majority of endogenously derived fatty acids are synthesized in the cytosol through a large polyfunctional enzyme in which each of the reactions is catalyzed by distinct domains (FAS I) [1]. Other processes which can contribute to cellular fatty acid synthesis are fatty acid chain elongation in the endoplasmic reticulum [2], and the recently identified mitochondrial fatty acid synthesis (FAS II) [3]–[8]. In this system, the required activities are catalyzed by separate enzymes and, therefore, the system resembles the well-characterized bacterial machinery [9].

Acyl carrier protein (ACP) of FAS II from mammalian mitochondria was isolated first as a component of complex I of the respiratory chain [10], but a soluble ACP pool has also been described [7]. Most of other FAS II components in mammals, including human, were originally identified *in silico* on the basis of amino acid sequence similarities with proteins characterized from other organisms such as prokaryotes, fungi or plants [3]–[5], [11]. Among the components which have been alighted on the basis of similarities to fungal proteins is 2-enoyl thioester reductase, which is also known as nuclear receptor binding factor 1, Nrbf1, or as mitochondrial enoyl-CoA reductase (Etr1/Mecr; EC 1.3.1.38). This enzyme catalyzes the reaction:
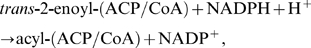
the last step of fatty acid synthesis spiral [4], [12]. The murine protein is encoded by *Mecr*, at CCDS18715 on chromosome 4, and the corresponding human gene *MECR* is located at CCDS30659 on chromosome 1.

To shed light on the function of the human mitochondrial FAS II, we used mice as a model system and report here generation of an artificial imbalance in the expression of *Mecr* when it is put under the control of the mouse metallothionein-1 (MT-1) promoter. The *Mecr* transgenic mice manifested focal mitochondrial clumping in myocardium, decreased cardiac mechanical function and decreased performance in the endurance tolerance test. Moreover, this work paves the road for identification of a novel set of genes whose improper expression could trigger development of cardiomyopathies.

## Results

**Figure 1:**
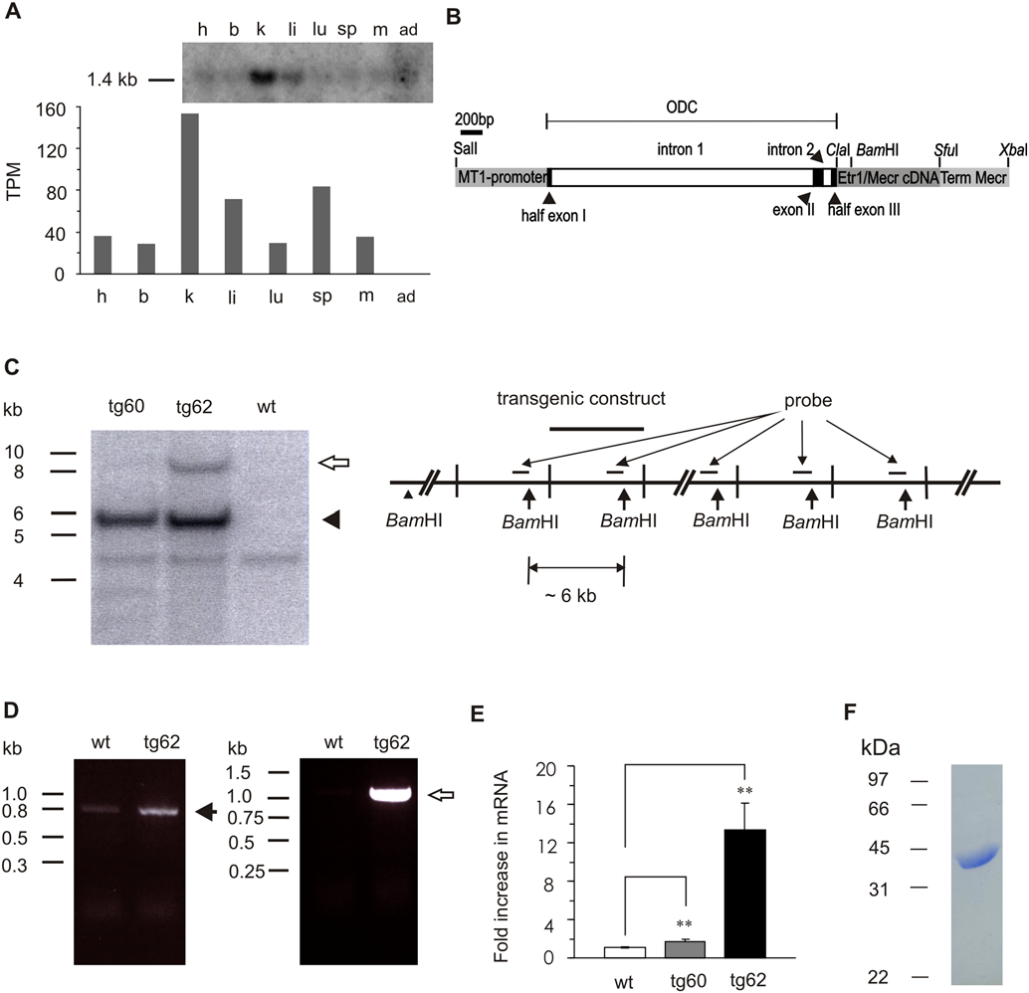
The generation of *Mecr* transgenic mice. A. Tissue expression pattern of *Mecr* in mice. The upper panel showed result of Northern blot. The lower panel showed the *in silico* analysis of *Mecr* expression pattern estimated from EST sources (UniGene, NCBI). The number indicated in y-axis refers to transcripts per million (TPM) that was calculated by the number of gene EST divided by totoal EST in pool of the indicated tissues. The symbol h, b, k, li, lu, sp, m, and ad refer to heart, brain, kidney, liver, lung, spleen, muscle, and adipose tissue, respectively. B. Transgenic construct consisting of the MT-1 promoter driving mouse *Mecr* expression; a 5′ noncoding region of the human *ODC* gene which includes the first two introns; full length mouse *Etr1/Mecr* cDNA including the translation initiation codon (ATG) and the stop codon in frame; followed by the 3′ UTR and termination signal of the mouse *Mecr* gene (Term. Mecr). C. Southern blot genotyping, schematic presentation of the integration of the *Mecr* transgenic construct into the mouse genome. Mouse genomic DNA was digested by *Bam*HI, giving a 6 kb fragment which is a marker for the transgene construct. The arrow head showed the size of the transgene construct (∼6 kb); the unfilled arrow shows an additional 9.5 kb fragment recognized by the probe only in tg62. The greater intensity of the 6 kb transgene band over that of the single-copy wild type band seen at approximately 4.3 kb in all three DNA samples demonstrates integration of multiple copies of the construct. The 9.5 kb signal arises from the 5′ end of the construct together with about 3.5 kb from the integration site. D. Reverse template PCR analysis of *Mecr* expression levels. Oligonucleotides which can recognize *Mecr* mRNA were used in left picture. The solid arrow shows an 800 bp band that was detected both in wild type and *Mecr* transgenic samples. Transgenic construct specific oligonucleotides were used in right picture. The unfilled arrow shows a 1.2 kb band detected only in *Mecr* transgenic samples. E. Real time PCR analysis of *Mecr* expression levels. Results are expressed as mean±SD (n = 4) **P<0.01 *Mecr* transgenic mice vs. wild type control. F. SDS-PAGE analysis of the purified His_6_-tagged Etr1/Mecr protein. The visualized band at 37 kDa corresponds to the predicted size of the peptide chain expressed from *E. coli*.

On-going research has revealed a number of mitochondrial processes whose existence has been recognized just recently. For instance, data coming from several laboratories indicate that mitochondria are capable of catalyzing *de novo* fatty acid synthesis [3]–[8]. Although experimental evidence shows that this conserved pathway is essential for mitochondrial respiratory function in fungi [12]–[14], its role in mammals has remained open. Here we describe the generation and analysis of mice overexpressing *Mecr* under the control of MT-1 promoter (Figure 1B). (*i*) *Mecr* was chosen because its gene product, Etr1/Mecr, is among the FAS II components which have been characterized best in mammals [4]. (*ii*) Mitochondrial respiratory function is highly dependent on its expression in the yeast model system [12], and (*iii*) MT-1 promoter is ubiquitously active in mice allowing sampling of multiple tissues for screening of responses in transgenic animals [15]–[17].

### Generation of *Mecr* transgenic mice

**Figure 2:**
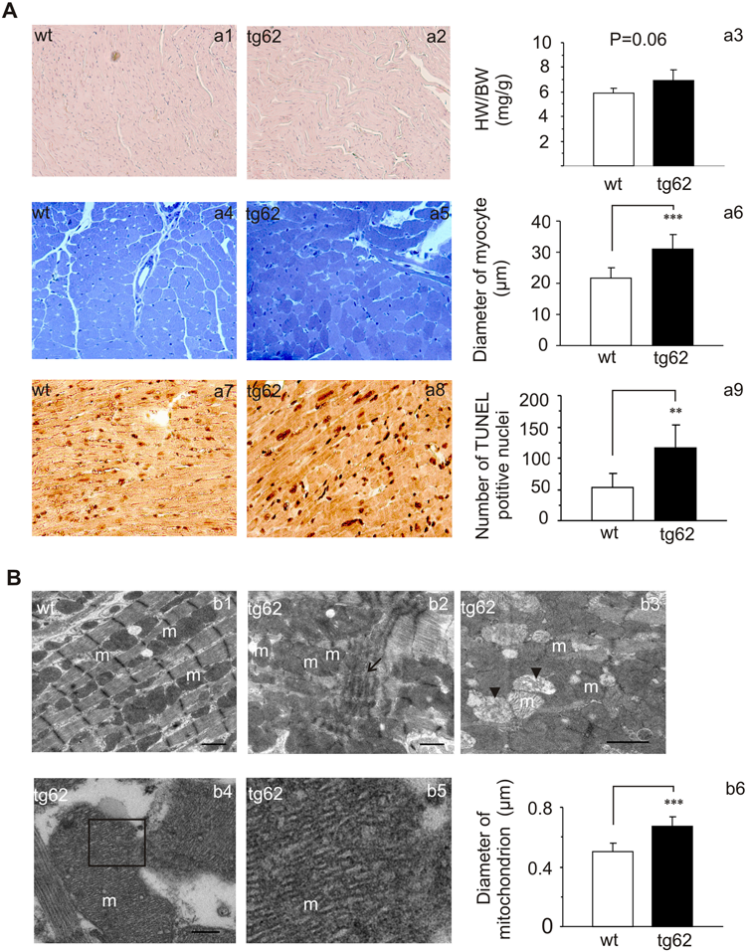
Histological analysis. A. Haematoxylin-eosin staining showed slightly distorted and wavy myofibers in *Mecr* transgenic hearts (a1 and a2). There is no significant difference in heart weight/body weight between wild type and *Mecr* transgenic mice (a3). Toluidine blue staining showed an uneven staining pattern in transgenic cardiomyocytes (a4 and a5). The diameter of myofibers was greater in transgenic heart than in wild type (SD (n = 6) ***P<0.001) (a6). TUNEL staining showed an increased number of positively stained nuclei (SD (n = 5) **P<0.005) in *Mecr* transgenic mice vs. controls (a7 through a9). B. Transmission electron micrographs show well-organized myofibrils and regularly located mitochondria in wild type hearts (b1), whereas in *Mecr* transgenic hearts myofibrils are disorganized (b2). Accumulated mitochondria and a few degenerating organelles can be also seen (b3). High-density cristae were only observed in transgenic mitochondria (b4). b5 shows an enlargement of the indicated area in b4. The average diameter of mitochondria is larger in *Mecr* transgenic mice than in wild type controls (SD (n = 6) ***P<0.001) (b6). Bars represent 1 µm (b1–b3) and 200 nm (b4), respectively. “m” represents mitochondrion. The arrows show distorted myofibrils; the arrow heads point to degenerated mitochondrion.

Two transgenic founders (tg60 and tg62) were generated which transmitted the transgene to their offspring in a Mendelian fashion. The different fragment sizes, visualized by Southern blotting, indicated that the transgenic construct was integrated into different locations of the genome in the tg60 and tg62 lines without any hint of multiple integrations (Figure 1C). When tissues from the *Mecr* transgenic mice lines were analyzed using histological approaches either by light or electron microscopies with morphological criteria, the affected organ was heart (Figure 2). In line with this, both lines of the *Mecr* transgenic mice displayed dysfunctional cardiac phenotype (see below). The *Mecr* in wild-type mice is expressed actively in kidney and liver, and moderately in heart and other tissues as shown by Northern blot and *in silico* analysis using data from UniGene database of the National Center for Biotechnology Information *(*NCBI*)* (Figure 1A).

Reverse template PCR showed that mRNA levels of *Mecr* was higher than wild type in heart muscle of both *Mecr* transgenic lines (Figure 1D). The increase was confirmed by semiquantitative real time PCR, the mRNA levels being 1.05±0.09 in wild type animals (wt) and 1.72±0.26 (*P*<0.01, vs. wt) and 13.3±2.8 (*P*<0.005, vs. wt) in tg60 and tg62, respectively (Figure 1E). When assayed, livers and muscle tissues from *Mecr* transgenic mice did not show significant increase of *Mecr* in mRNA levels. To test whether the overexpression construct was properly transcribed and processed, cDNA for artificial *Mecr* was prepared from tg62, the nucleotide sequence was checked, the cDNA was subcloned into the pET3a vector and expressed in BL21(DE3) pLysS cells. The soluble protein supernatant of the cell lysate was subjected to ADP-Sepharose chromatography, and the fractions showing Etr1/Mecr activity were analyzed by SDS-PAGE and mass spectrometry. A positive band from the active fractions was confirmed by mass spectrometry to be Etr1/Mecr. To confirm this further, the artificial cDNA was cloned into pET23a vector containing a C-terminal His_6_-tag and the tagged Etr1/Mecr variant was purified to homogeneity using Ni-NTA and size exclusion chromatography columns (Figure 1F). The purified tagged Etr1/Mecr was enzymatically active when tested with C4 - C16 2-enoyl-CoA substrates. These results demonstrated that the *Etr1/Mecr* transgene mRNA is properly processed and encodes enzymatically active protein.

However, the demonstration of increased Etr1/Mecr in *Mecr* transgenic hearts failed based on both immunoblot analysis and enzyme activity assays, for discussion, see below. Administration of zinc either by adding 25 mmol/L ZnSO_4_ to drinking water (tested for 1 week and 1 month) or by *i.p.* injection with ZnSO_4_·7H_2_O (20 mg/kg, animals were sacrificed 24 h after injection as described in references [18], [19], gave no further increase in *Etr1/Mecr* mRNA in either of the *Mecr* transgenic line. Therefore, this study was continued without administration of zinc to animals.

The weight gain and litter sizes of *Mecr* transgenic mice did not differ significantly from wild type controls. The life span of the transgenic mice was not different from that of wild type littermates. The expression level of various heart failure mark genes [20], e.g., myosin heavy chain (αMHC), sarcoplasmic or endoplasmic reticulum calcium adenosine triphosphatase 2 (SERCA2), atrial natriuretic peptide (ANP), brain natriuretic peptide (BNP), and relevant transcription factors, e.g., nuclear respiratory factor-1 (NRF1) [21], peroxisome proliferator-activated receptor γ coactivator 1-α (PGC-1α) [22], peroxisome proliferator-activated receptor (PPARα) [22], estrogen-related receptor α (ERRα) [21], were monitored using semiquantitative real time PCR. The mRNA levels of ANP, BNP, ERRα, αMHC, NRF1, PGC-1α, PPARα, and SERCA2 were 1.29±0.25, 1.00±0.23, 1.15±0.45, 0.85±0.43, 1.09±0.36, 0.91±0.22, 0.94±0.22, and 0.97±0.21, respectively (n = 5, n.s.), when the mRNA levels in the wild type animals were set to 1.0.

### Morphology of heart and mitochondria

In haematoxylin-eosin staining, transgenic hearts, when compared to wild type, showed slightly distorted myofibers (Figure 2A, a1 and a2). However, the heart weight/body weight of transgenic mice did not differ significantly from that of wild type controls (Figure 2A, a3). In toluidine blue sections taken from plastic embedded material, cardiomyocytes in transgenic hearts displayed a mosaic staining whereas the cells in wild type controls showed even staining (Figure 2A, a4 and a5). The average diameter of myocytes was 21.8±3.1 µm for wild type and 31.1±4.5 µm for tg62 (*P*<0.001). TUNEL staining showed an increased number of positive nuclei in transgenic hearts (52±24 for wild type vs. 106±38 for tg62 per 10 high-power fields; *P*<0.005) (Figure 2A, a7 through a9).

Transmission electron microscopy showed focal disorganizations of myofibrils and aberrant mitochondria in transgenic hearts (Figure 2B, b1 and b2). Furthermore, the mitochondria in transgenic hearts appeared to be densely packed, so that even parts of the outer membranes were sometimes fused; also, degenerating swollen mitochondria were often detected (Figure 2B, b3). Especially in large mitochondria, the cristae density was high when compared to wild type organelles (Figure 2B, b4 and b5). The average diameter of mitochondria was enlarged in transgenic hearts (0.51±0.05 µm for wild type and 0.68±0.06 µm for tg62, *P*<0.001) (Figure 2B, b6). Mice from both tg60 and tg62 lines showed similar phenotype in histological analysis.

### Cardiac function *in vivo*

**Figure 3:**
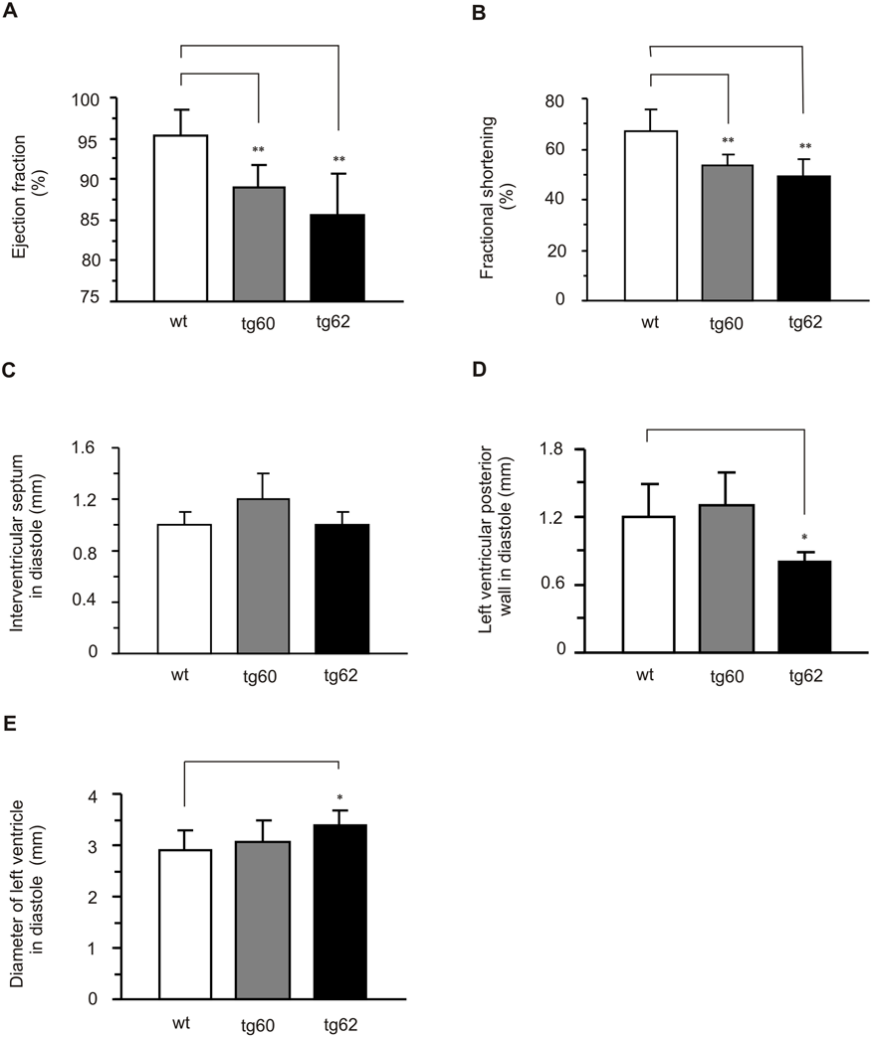
Echocardiography of wild type, *Mecr* transgenic line tg60, and *Mecr* transgenic line tg62. A. Ejection fraction; B. Fractional shortening; C. Interventricular septum in diastole; D. Left ventricular posterior wall in diastole; E. Diameter of left ventricle in diastole. Results are expressed as mean±SD (wt n = 9; tg60 n = 4; tg62 n = 5). *P<0.05, **P<0.01, *Mecr* transgenic mice vs. wild type controls.

Cardiac function was evaluated using echocardiography *in vivo*. Both female and male *Mecr* transgenic mice showed similar impaired cardiac phenotype compared to their wild type littermates (data not shown). Female mice were chosen for further studies. Both tg60 and tg62 transgenic lines had significantly decreased cardiac systolic function (Figure 3A and 3B). In particular, the ejection fraction was reduced in *Mecr* transgenic mice (95.4±3.2% in wt vs. 88.1±3.3% in tg60 and 85.3±3.9% in tg62; *P*<0.01) (Figure 3A) and fractional shortening was reduced as well (66.8±8.7% in wt vs. 52.2±4.6% in tg60 and 48.5±4.5% in tg62; *P*<0.01) (Figure 3B). The thickness of the interventricular septum did not significantly differ between groups showing that there was no hypertrophy present in *Mecr* transgenic mice (Figure 3C). In support of this, the thickness of the left ventricular posterior wall was even decreased in tg62 (1.2±0.3 mm in wt vs. 0.8±0.1 mm in tg62; *P*<0.05) (Figure 3D). Noteworthily, in the tg62 (high expression), but not in the tg60 (low expression) transgenic line, left ventricular dilatation was seen, as the diameter of the left ventricle was significantly greater than in wild type (2.9±0.4 mm in wt vs. 3.1±0.4 mm in tg60, p = 0.47 and 3.4±0.3 mm in tg62, *P*<0.05) (Figure 3E).

Left ventricular mass measured by echocardiography was 120±11 mg in wild type mice and 164±17 mg in *Mecr* transgenic mice (n.s.). The decreased systolic function and left ventricular dilatation without any signs of hypertrophy underlines the importance of *Mecr* in dilatative rather than hypertrophic processes in the heart.

### Mechanical function of isolated perfused heart

**Figure 4:**
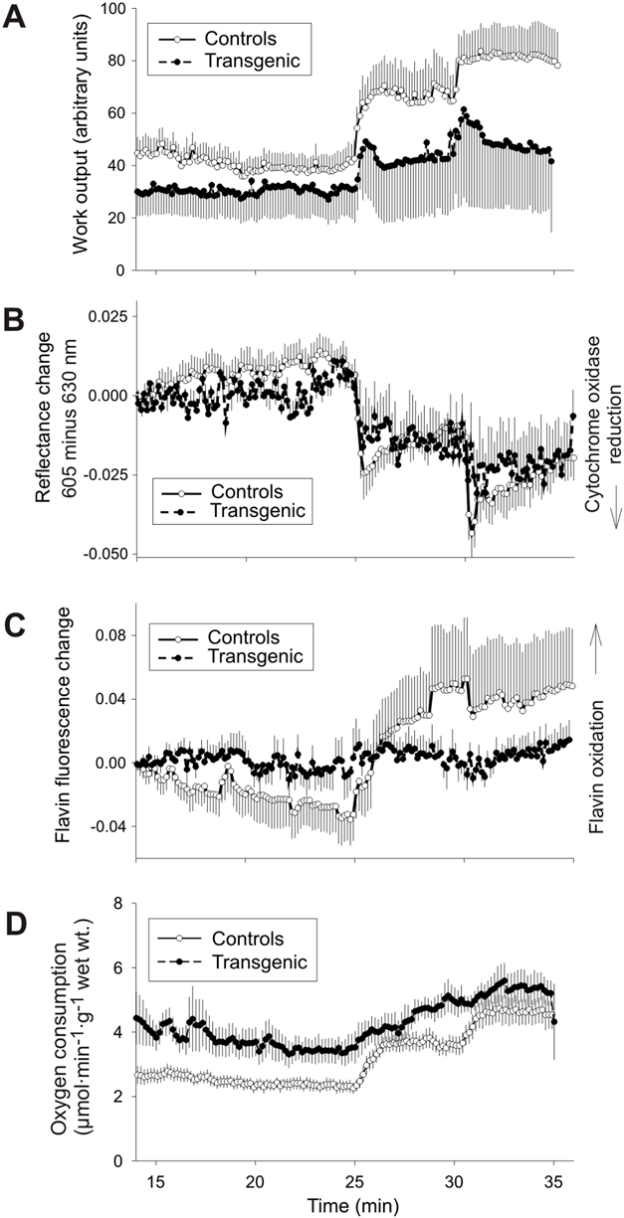
Changes in work output, redox state of mitochondrial respiratory chain, oxygen consumption, and heart rate of isolated perfused hearts from wild type and *Mecr* transgenic mice. From the 25 min time point, the hearts were electrically paced to 5 Hz and from the 30 min time point they were paced to 7.5 Hz. A. The heart-rate-related work output changes are significant in the wild type hearts (*P* = 0.011), whereas they are not in the *Mecr* transgenic hearts (*P* = 0.737). B. Redox changes of cytochrome oxidase as measured by reflectance spectrum changes. A reflectance decrease indicates a shift of the redox state towards reduction. The heart rate-related change in the redox state of cytochrome oxidase is significant in both groups (*P*<0.001). C. Flavoprotein redox state as measured by means of flavin fluorescence. An increase in fluorescence indicates oxidation. D. Oxygen consumption. The curves represent means±SD from 5 and 8 independent experiments on *Mecr* transgenic and control hearts, respectively.

The hearts from *MECR* transgenic mice and wild type controls were subjected to retrograde perfusion according to the Langendorff procedure [23]. Values from the two *MECR* transgenic groups did not differ significantly and were therefore pooled. The mechanical work output of the hearts was monitored at three workload levels: at their endogenous beating frequency (3.2–3.8 Hz), pacing at 5 Hz, and pacing at 7.5 Hz. Work output was given as the product of dPmax/dt and heart rate in arbitrary units. At the endogenous frequency, the average work output for the 23–24 min perfusion interval was not significantly different in *MECR* transgenic hearts (36.2±11.6) from that of wild-type controls (38.3±5.9), although the heart rate tended to be slower in the transgenic hearts (2.95 Hz±0.70, mean±SD) than in controls (3.43±0.69, mean±SD) (Figure 4A). At the paced beating frequency of 5 Hz (27–29 min), average work output was also slightly lower in the *MECR* transgenic hearts (55.3±24.6) than in controls (65.6±11.8) (Figure 4A). At 7.5 Hz (32–34 min), the work output was 58.0±25.1 in the *MECR* transgenic hearts and 81.9±10.2 in controls (Figure 4A). In the wild type hearts there was a linear correlation (*P*<0.005) between heart rate and work output, whereas the *MECR* transgenic hearts did not increase their work output in proportion to pacing frequency. When the pacing was increased to 10 Hz, the wild type hearts responded, but the *MECR* transgenic hearts did not (data not shown).

### Cellular redox state and oxygen consumption

The mitochondrial redox state was monitored at two levels of the respiratory chain, the cytochrome c oxidase and the free NADH/NAD^+^ pool. The redox state of cytochrome *c* oxidase, the terminal oxidase of the mitochondrial respiratory chain, was monitored by reflectance spectrum changes. A shift towards reduction was seen upon increase in the workload, in agreement with the current view of regulation of mitochondrial respiration (Figure 4B), and the effect of the pacing frequency change was smaller in the *MECR* transgenic hearts than in wild type.

The mitochondrial matrix NADH/NAD^+^ ratio can be monitored in a compartment-specific manner by recording flavoprotein fluorescence signal, which is mainly contributed by the lipoamide dehydrogenase of the mitochondrial oxoacid dehydrogenases and is in equilibrium with the NADH/NAD^+^ pool [24]. It was found that an increase in workload caused an increase in flavin fluorescence, indicating decrease in the NADH/NAD ratio (Figure 4C), and the effect of workload increase was smaller in the *MECR* transgenic hearts.

Oxygen consumption increased upon increase in the workload in all experimental groups. The *MECR* transgenic hearts differed from the controls in that the increase upon heart rate increase from 5 to 7.5 Hz was smaller, although this is in accord with the smaller increase in work output due to an increase in the pacing frequency from 5 to 7.5 Hz (Figure 4D).

The CrP/Pi ratio in the *MECR* transgenic hearts (1.26±0.17) (mean±SD) after pacing at 7.5 Hz tended to be lower than that in controls (1.40±0.43). Experimental variation was large so the difference did not reach statistical significance. The CrP/Pi ratio is a parameter well suited to describing the cellular energy state in tissues containing creatine kinase, since the changes in the CrP and Pi concentrations are usually reciprocal.

The mitochondria were isolated from mouse livers and hearts, and the rates of the state 3 and 4 respiration were monitored in the presence of various substrates. There were no significant differences in the respiratory rates between the mitochondria from wild-type and transgenic mice livers and hearts (Table S1). The results suggest that the decreased physiological performance as detected *ex vivo* (Figure 4) might not result from general impairment of mitochondrial function, but rather from uneven distribution of dysfunctional mitochondria in myocardial cells in *Mecr* transgenic mice. This suggestion is also in line with observations visualized by electron microscopy (Figure 2B).

### Exercise endurance test

**Figure 5:**
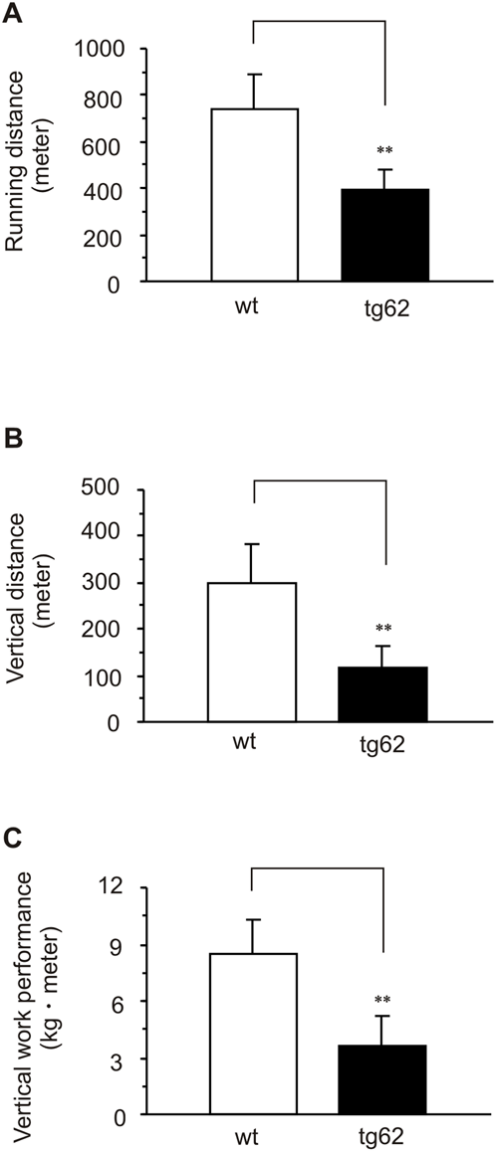
The Running endurance exercise performance. Performance in the exercise tests as indicated by A. running distance, B. vertical distance, C. vertical work performance. Results are expressed as mean±SD **P<0.01, *Mecr* transgenic mice vs. wild type control.

Exercise intolerance is one of the key symptoms of dilated cardiomyopathy. Because *Mecr* transgenic mice expressed a phenotype with some features of dilated cardiomyopathies, we challenged the mice to exercise on a treadmill. Three parameters were monitored: running distance, vertical distance and vertical work performance. Treadmill values for wild type mice (value for tg62 in parenthesis) for running distance were 740±150 m (396±85 m, *P*<0.005), for vertical distance were 301±81 m (118±43 m, *P*<0.005) and for vertical work performance were 8.45±1.85 kg m (3.62±1.62 kg m, *P*<0.005) (Figure 5A through 5C). When tested separately, animals from the tg60 line also showed poor performance in the exercise test.

## Discussion

Phenotype analysis of the mice overexpressing *Mecr* revealed the existence of a novel link between expression of FAS II genes, mitochondria and mechanical function in myocardium tissue. Northern blot analysis of samples from various mammalian tissues has shown that heart muscle is among the tissues showing high mRNA levels for genes of FAS II [3], [4], [8], [11]. The overexpression of *Mecr* in mice resulted in development of myocardial dysfunction as demonstrated by several lines of evidence. The *Mecr* transgenic animals have impaired mechanical heart function *in vivo* as documented by (*i*) echocardiography and (*ii*) decreased physical performance in exercise endurance tests. (*iii*) In perfusion experiments, the isolated hearts from transgenic mice did not increase work output linearly upon increasing the beating rate by electric pacing whereas this correlation was linear with hearts from wild type mice. (*iv*) Both light and electron microscopy showed disorganization in cardiac myofibrils. Transmission electron microscopy further revealed a high density of cristae and focal clumping of mitochondria in myocardium.

To rule out the possibility that the observed phenotypes are due to construct-dependent interruption of endogenous genes involved in heart function, we analyzed two independent transgenic mouse lines using different approaches. Southern blotting proved that the transgenes were incorporated in a line-specific manner and that different numbers of copies were inserted into distinct but not multiple sites in the genome (Figure 1C). It is unlikely that construct integration into different loci of mouse genome *per se* would confer the same phenotype. Therefore, the findings favor the view that the phenotype of the transgenic mice resulted from the overexpression of the construct rather than from interruption of an endogenous gene.

Echocardiographic monitoring of cardiac function *in vivo* showed that ejection fraction and fractional shortening were reduced in *Mecr* transgenic mice. Additionally, these mice showed a tendency to have increased left ventricular mass and dilatation of the left ventricle, suggesting a dilatative rather than a hypertrophic process behind the impaired function. This was corroborated in isolated perfused hearts, which showed decreased work output in the *Mecr* transgenic hearts. When the pacing was increased to 10 Hz, the wild type hearts responded, but many of the *Mecr* transgenic hearts failed to follow the pacing. Noteworthily, the levels of mRNAs of heart failure mark genes and their relevant transcription factors did not show significant changes in *Mecr* transgenic hearts compared to wild type controls.

The *Mecr* transgenic mice showed diminished performance in the endurance test, resembling one of the hallmark symptom of patients with dilatative cardiomyopathy [25]. The running distance of the *Mecr* transgenic mice before fatigue is about half of that of wild type controls. It is worth noting that transmission electron microscopy of skeletal muscle did not reveal clumping of mitochondria nor was myofibrillar disorganization detected.

Haematoxylin-eosin staining and transmission electron micrographs showed focal distortion of myofibers in the *Mecr* transgenic mouse hearts, accompanied by aggregation and an unusual ultrastructure of the mitochondria. The increased density of cristae that was observed in the heart mitochondria of *Mecr* transgenic mice resembles a feature of mitochondria from skeletal muscles found in Luft disease [26]. Significantly, the average size of mitochondria from *Mecr* transgenic hearts is greater than that of controls (Figure 2B, b6). The morphological changes of *Mecr* transgenic mitochondria may be partially responsible for altered oxygen consumption and function of the transgenic hearts upon challenging as vocalized by heart perfusion experiments. Thus, the mitochondrial phenotype observed previously in yeast upon overexpression of *Etr1* [12] , can be reproduced in mice, at least in part, demonstrating conserved regulatory mechanisms. The mitochondrial swelling in yeast is not due to the loading of yeast mitochondria with Etr1p, but requires an unidentified signal from the mitochondria to the nucleus [12], [27].

In bacteria and in the plastids of plants, FAS II serves as the major route for generation of cellular fatty acids [9], whereas in yeast and mammals, the end-products of the mitochondrial FAS system constitute only a small fraction of cellular fatty acids and their nature has not yet been identified conclusively. The proposed products include an long-chain acyl groups for remodeling of cardiolipins and octanoyl group for lipoic acid synthesis [13], [28]. Cardiolipins are acidic phospholipids found mainly in the mitochondrial inner membrane in mammals [29]. Even though lipoic acid is regarded as a fat soluble vitamin, mammalian mitochondria also contain lipoic acid synthase [30]. In cells, lipoic acid is covalently attached to the E2 subunits of mitochondrial α-ketoacid dehydrogenase complexes [31]. Recently a mouse model carrying disruption of *Lias*, gene encoding lipoic acid synthase, has been described [31]. Interestingly, this disruption resulted in embryonic lethality due to failure in development of haematopoietic and cardiovascular system.

There may be several reasons for our failure to detect overexpression of *Etr1/Mecr* at the protein level using either whole heart preparations or isolated mitochondria. The amount of endogenous Etr1/Mecr in mitochondria is very low [4], and the fact that we had the antibody to human but not to mouse protein might make its detection more difficult. Another similar example is *Aurora A* transgenic mice, when Aurora A protein is overexpressed, it is ubiquitinated by the cells, which targets it toward the ubiquitin-linked protein degradation system. Thus, overexpression can only be detected at the mRNA level [32].

The rather similar phenotype of tg60 and tg62 mice lines raises a possibility that *Mecr* expression is controlled post-transcriptionally either at translational or post-translational levels. These options are currently under investigation in our laboratory. Interestingly, it has been reported that mice transgenic for the MT-1-driven tpr-met oncogene develop a clear tumor phenotype, while the overexpression is detectable neither at the mRNA level nor at the protein level in any tissues except tumors [33].

Our transgenic mice resemble to some extent moderately overexpressing caspase-8 transgenic mice [34], whose survival rates do not significantly differ from wild type mice and which developed a dilated heart and decreased systolic function. We are on the way to generate *Mecr* cardiac specific expression transgenic mice as well as *Mecr* knockout mice. When these transgenic lines are available, we are going to addresses several issues: (*i*) how the expression levels of *Mecr* are regulated *in vivo*; (*ii*) the molecular and physiological mechanisms of the cardiac phenotype of the *Mecr* transgenic mice; (*iii*) whether the poor exercise performance of the *Mecr* transgenic mice is solely because of the overexpression of *Mecr* in cardiac myocytes.

Based upon the data we obtained from various independent experiments, we conclude that the *Mecr* transgenic mice suffer from a heart dysfunction which is obviously linked to an impairment in mitochondrial function. This is in line with reports suggesting that there exists a correlation between mitochondrial dysfunction and contractile dysfunction of heart [35]. We expect that the *Etr1/Mecr* transgenic mice could provide a research model to study the physiological function of the mitochondrial FAS II in mammals.

## Materials and Methods

Two lines of *Mecr* transgenic mice were generated and their expressions are under control of MT-I promoter. PCR and southern blotting were used for genotyping. Reverse template RCR and Semiquantitative real time PCR were applied to check the expression level of *Mecr* and other mark genes. Male animals at the age of 1–1.5 years were used if not otherwise stated. Notably, similar phenotypic changes were also observed in younger mice (e.g., at the age of 6–7 months). All experiments were performed in accordance with regulations formulated by the Animal Care and Use Committee of Oulu University.

### Southern blot

Mouse genomic DNA was digested by *Bam*HI. There is a single cutting site within the overexpression construct at 4.2 kb/6 kb (6 kb is the size of full length construct.) (Figure 1B). The probe (1 kb) was made by digestion of the construct with *Pvu*II; there are two *Pvu*II sites, at 3.3 kb and 4.3 kb, within the construct. The intensity of the 6 kb band is proportional to the copy number of the transgene.

### Northern blot

Total RNA Northern Blot was ordered from USBiological (Swampscott, MA). The probe (0.8 kb) was made by digestion of the *Mecr* cDNA with *Bam*HI and *Not*I. The experiments were carried out according to supplied manual.

### Reverse template RCR and Semiquantitative real time PCR

The cDNAs were produced with a First Strand cDNA Synthesis Kit (MBI Fermentas, Heidelberg, Germany) using total RNA isolated from mouse heart and liver samples. Semiquantitative real time PCR was performed with a 7500 Real Time PCR System (Applied Biosystems, FosterCity, CA) using fluorogenic probe-based TaqMan chemistry (Applied Biosystems, FosterCity, CA), using GAPDH as an internal control for normalizing the gene expression.

### Overexpression and purification of Etr1/Mecr

cDNA of *Mecr* was obtained by reverse template PCR (MBI Fermentas, Heidelberg, Germany) using oligos that can only recognize artificial *Mecr* sequence from tg62 line and cloned into pET3a and pET23a vectors. The overpression, purification of His-tagged protein and enzymatic assays were carried out using the protocol as described in [36].

### Histological analysis

The heart tissue samples were fixed in phosphate buffered 4% paraformaldehyde and embedded in paraffin, and 4 µm sections were stained with haematoxylin-eosin according to standard protocols. TUNEL staining was done according to the instructions provided with the *in situ* cell death detection kit (Roche Diagnostics, Mannheim, Germany). The sections were examined using an Olympus BX50 light microscope and analyzed using software supplied (Soft Imaging Systems, Munster, Germany).

For transmission electron microscopy (TEM), tissue samples from mouse heart were fixed in 1% glutaraldehyde and 4% formaldehyde in 0.1 M phosphate buffer, pH 7.2, and then dehydrated in acetone and embedded in Embed 812 (Electron Microscopy Sciences, Fort Washington, PA). Thin sections of 80 nm were cut by a Leica Ultracut UCT ultramicrotome and examined in a Philips CM100 transmission electron microscope.

### Echocardiographic analysis

The cardiac function of both male and female mice was evaluated using echocardiography. The dimensions of cardiac chambers and left ventricular function were assessed using transthoracic echocardiography with an Acuson Ultrasound System (Sequoia 512) and a 15-MHz linear transducer (15L8). Ketamine (50 mg/kg IP) and xylazine (10 mg/kg IP) were used for anesthesia. A short-axis view of the left ventricle at the level of the papillary muscles was obtained by using 2D imaging for the M-mode recording. Left ventricular end-systolic (LVESD) and end-diastolic (LVEDD) dimensions as well as thickness of interventricular septum (IVS) and posterior wall (PW) were measured from the M-mode tracings. LV fractional shortening (LVFS) and ejection fraction (EF) were estimated from the M-mode LV dimensions by applying the following formulas: LVFS (%) = [(LVEDD−LVESD)/LVEDD]×100 and EF (%) = [(LVEDD*^3^*−LVESD*^3^*)/LVEDD*^3^*]×100.

### Heart perfusion

The mice were sacrificed by decapitation after cervical dislocation. The aorta was immediately cannulated and perfusion commenced *in situ* with ice-cold perfusion medium. The heart was then dissected out and connected to the perfusion apparatus. The perfusion fluid was Krebs-Henseleit perfusion medium containing 118.5 mmol/L NaCl, 4.7 mmol/L KCl, 2.5 mmol/L CaCl_2_, 0.25 mmol/L Ca-EDTA, 1.2 mmol/L MgSO_4_, 1.2 mM KH*_2_*PO_4_, 25 mmol/L NaHCO_3_, 10 mmol/L glucose. The hearts were then perfused in a modified Langendorff apparatus with a pressure of 100 cm H*_2_*O (9.81 kPa) and with the medium maintained by a thermostat at 37°C and with an O*_2_*∶CO*_2_* ratio of 19∶1. The heart rate, left ventricular systolic pressure, coronary flow, and oxygen consumption were measured. There was a stabilization period of at least 30-minutes before electrical pacing with an epicardial Ag/AgCl electrode was begun at a voltage 50% above the threshold. All experiments ended with freeze-clamping of the heart.

The heart was enclosed in a thermostated, light-tight chamber equipped with fiber optics for epicardial readout of reflectance spectrum changes (605 nm versus 630 nm for cytochrome *c* oxidase) and fluorescence (emission above 510 nm with 465-nm excitation for flavin) by means of a three-channel spectrophotometer-fluorometer and the data collected via DAQ-card (National Instruments, Austin, TX) and stored on a PC at 2-s intervals. Left ventricular pressure was monitored through the ventricular wall by inserting a saline-filled Teflon cannula connected to a Statham P231D pressure transducer linked to a Statham SP1400 pressure monitor. The pressure wave signal was fed to a Lab-PC+ data acquisition card (National Instruments, Austin, TX) and heart function parameters (heart rate, peak systolic pressure, diastolic pressure and peak pressure development) were calculated on-line with custom designed software and stored on a computer at 4-s intervals. Coronary flow was measured during the experiments by using a drop counter with an analog frequency output. Oxygen consumption was calculated from the arteriovenous concentration difference multiplied by coronary flow.

### Metabolite assays

The heart was quick-frozen with aluminum clamps precooled in liquid nitrogen, and stored in liquid N_2_ until processed further. The frozen heart tissue was immersed in 8% (wt/vol) HClO_4_ in 40% (vol/vol) ethanol precooled to −20°C, and homogenized immediately with an Ultra-Turrax homogenizer (Ika Werk, Midland, ON, Canada). After centrifugation the supernatant was neutralized with 3.75 mol/L K*_2_*CO*_3_* containing 5 mol/L triethanolamine hydrochloride [37].

Creatine phosphate (CrP), creatine (Cr) and inorganic phosphate levels were measured in the supernatant by enzymatic assays [37], [38]. The appearance or disappearance of NADH was measured in an Aminco DW-2 dual-wavelength spectrophotometer using an ε*_340_* minus ε*_385_* value of 5.33 L/mmol per centimeter [38], [39].

### Measurements of mitochondrial O_2_ consumption

Rates of oxygen consumption in the presence of various substrates were measured polarographically with the Rank Brothers oxygen electrode (Bottisham, Cambridge, UK). The incubation medium contained 130 mM KCl, 10 mM HEPES, 5 mM potassium phosphate, 0.1 mM EGTA, 1 mg/ml bovine serum albumin (essentially fatty acid free) and 2 mM MgCl_2_, pH 7.2. The incubations contained 0.5–1.2 mg/ml of mitochondrial protein in a final volume of 0.7 ml at 30°C, and substrates as given.

### Exercise performance test

The animals were subjected to exercise performance tests. The previous protocol [40] was revised slightly with respect to speed and inclination. Two days before the exercise performance test, all mice were familiarized with running on a motorized running belt with rotating flexible plastic strips at the rear of the treadmill to prod the mice to run. 10 min exercise with a treadmill inclination of 9° was used for the familiarization run. Treadmill speed was 9 m/min on the first day and 11 m/min on the second day. Before the final test, mice were placed on the belts and allowed to adapt to the surroundings for 5 min before running. The treadmill was started at a speed of 9 m/min with a 0° slope for 9 min. Then the speed and inclination were raised to 11 m/min and 6°, respectively. Speed was increased by 1–3 m/min every 3 min to a maximum of 28 m/min, and the inclination gradually increased by 3° every 9 min to a maximum of 12°. Exercise was stopped when mice were unable to maintain running despite repeated contact with the rotating plastic strips. Each mouse was then immediately returned to its home cage from the treadmill. Exercise performance was assessed from the performance tests based on the following three parameters: the duration of the run (in minutes), the distance run (in meters), and vertical work performance (in kg•m). Vertical work performance was calculated as the product of body weight (kg) and vertical distance (meters), where vertical distance equals distance run × sinα, where α is the slope of the treadmill from 0° to 12°.

### Statistical Analysis

Values are expressed as mean±standard deviation (SD) and analyzed by the Student's *t* test for detecting significant differences. The haemodynamic variables were analyzed with 2-way repeated-measures ANOVA. Echocardiographic measurements were analyzed using normalized values with 1-way ANOVA followed by the Student-Newman-Keul *post hoc* test. A value of *P*<0.05 was considered statistically significant.

## Supporting Information

Table S1 The rates of state 3 and 4 respiration of liver and heart mitochondria with various substrates. The liver and heart mitochondria were isolated as described [1] after 12 h fast. The incubations were carried out with 0.5–1.2 mg/ml of mitochondrial protein at 30°C in the appropriate medium (Materials and Methods) and 2 mM glutamate, 2 mM malate, 2 mM pyruvate, or 2 mM succinate. ADP was used at 1 mM concentration. The rate of oxygen consumption (nmol O2/min per mg protein) was monitored with Clark type oxgen electrode. The results are expressed as mean±standard deviation. The statistical significance was estimated using the Student's t test and the P-values are given.
(0.02 MB DOC)

## References

[1] Smith S (1994). The animal fatty acid synthase: one gene, one polypeptide, seven enzymes.. Faseb J.

[2] Denic V, Weissman JS (2007). A molecular caliper mechanism for determining very long-chain fatty acid length.. Cell.

[3] Joshi AK, Zhang L, Rangan VS, Smith S (2003). Cloning, expression, and characterization of a human 4′-phosphopantetheinyl transferase with broad substrate specificity.. J Biol Chem.

[4] Miinalainen IJ, Chen ZJ, Torkko JM, Pirila PL, Sormunen RT (2003). Characterization of 2-enoyl thioester reductase from mammals. An ortholog of YBR026p/MRF1'p of the yeast mitochondrial fatty acid synthesis type II.. J Biol Chem.

[5] Zhang L, Joshi AK, Smith S (2003). Cloning, expression, characterization, and interaction of two components of a human mitochondrial fatty acid synthase. Malonyltransferase and acyl carrier protein.. J Biol Chem.

[6] Zhang W, Crocker E, McLaughlin S, Smith SO (2003). Binding of peptides with basic and aromatic residues to bilayer membranes: phenylalanine in the myristoylated alanine-rich C kinase substrate effector domain penetrates into the hydrophobic core of the bilayer.. J Biol Chem.

[7] Cronan JE, Fearnley IM, Walker JE (2005). Mammalian mitochondria contain a soluble acyl carrier protein.. FEBS Lett.

[8] Autio KJ, Kastaniotis AJ, Pospiech H, Miinalainen IJ, Schonauer MS (2008). An ancient genetic link between vertebrate mitochondrial fatty acid synthesis and RNA processing.. Faseb J.

[9] White SW, Zheng J, Zhang YM, Rock (2005). The structural biology of type II fatty acid biosynthesis.. Annu Rev Biochem.

[10] Runswick MJ, Fearnley IM, Skehel JM, Walker JE (1991). Presence of an acyl carrier protein in NADH:ubiquinone oxidoreductase from bovine heart mitochondria.. FEBS Lett.

[11] Zhang L, Joshi AK, Hofmann J, Schweizer E, Smith S (2005). Cloning, expression, and characterization of the human mitochondrial beta-ketoacyl synthase. Complementation of the yeast CEM1 knock-out strain.. J Biol Chem.

[12] Torkko JM, Koivuranta KT, Miinalainen IJ, Yagi AI, Schmitz W (2001). Candida tropicalis Etr1p and Saccharomyces cerevisiae Ybr026p (Mrf1'p), 2-enoyl thioester reductases essential for mitochondrial respiratory competence.. Mol Cell Biol.

[13] Schneider R, Massow M, Lisowsky T, Weiss H (1995). Different respiratory-defective phenotypes of Neurospora crassa and Saccharomyces cerevisiae after inactivation of the gene encoding the mitochondrial acyl carrier protein.. Curr Genet.

[14] Schneider R, Brors B, Burger F, Camrath S, Weiss H (1997). Two genes of the putative mitochondrial fatty acid synthase in the genome of Saccharomyces cerevisiae.. Curr Genet.

[15] Alhonen L, Heikkinen S, Sinervirta R, Halmekyto M, Alakuijala P (1996). Transgenic mice expressing the human ornithine decarboxylase gene under the control of mouse metallothionein I promoter.. Biochem J.

[16] Sandgren EP, Luetteke NC, Palmiter RD, Brinster RL, Lee DC (1990). Overexpression of TGF alpha in transgenic mice: induction of epithelial hyperplasia, pancreatic metaplasia, and carcinoma of the breast.. Cell.

[17] Behringer RR, Cate RL, Froelick GJ, Palmiter RD, Brinster RL (1990). Abnormal sexual development in transgenic mice chronically expressing mullerian inhibiting substance.. Nature.

[18] Baggio L, Adatia F, Bock T, Brubaker PL, Drucker DJ (2000). Sustained expression of exendin-4 does not perturb glucose homeostasis, beta-cell mass, or food intake in metallothionein-preproexendin transgenic mice.. J Biol Chem.

[19] Suppola S, Pietila M, Parkkinen JJ, Korhonen VP, Alhonen L (1999). Overexpression of spermidine/spermine N1-acetyltransferase under the control of mouse metallothionein I promoter in transgenic mice: evidence for a striking post-transcriptional regulation of transgene expression by a polyamine analogue.. Biochem J.

[20] Boluyt MO, Robinson KG, Meredith AL, Sen S, Lakatta EG (2005). Heart failure after long-term supravalvular aortic constriction in rats.. Am J Hypertens.

[21] Finck BN, Kelly DP (2007). Peroxisome proliferator-activated receptor gamma coactivator-1 (PGC-1) regulatory cascade in cardiac physiology and disease.. Circulation.

[22] Finck BN (2007). The PPAR regulatory system in cardiac physiology and disease.. Cardiovasc Res.

[23] Chain EB, Mansford KR, Opie LH (1969). Effects of insulin on the pattern of glucose metabolism in the perfused working and Langendorff heart of normal and insulin-deficient rats.. Biochem J.

[24] Hassinen I, Chance B (1968). Oxidation-reduction properties of the mitochondrial flavoprotein chain.. Biochem Biophys Res Commun.

[25] Coats AJ (2001). Exercise and heart failure.. Cardiol Clin.

[26] Luft R, Ikkos D, Palmieri G, Ernster L, Afzelius B (1962). A case of severe hypermetabolism of nonthyroid origin with a defect in the maintenance of mitochondrial respiratory control: a correlated clinical, biochemical, and morphological study.. J Clin Invest.

[27] Kastaniotis AJ, Autio KJ, Sormunen RT, Hiltunen JK (2004). Htd2p/Yhr067p is a yeast 3-hydroxyacyl-ACP dehydratase essential for mitochondrial function and morphology.. Mol Microbiol.

[28] Witkowski A, Joshi AK, Smith S (2007). Coupling of the de novo fatty acid biosynthesis and lipoylation pathways in mammalian mitochondria.. J Biol Chem.

[29] Schlame M, Shanske S, Doty S, Konig T, Sculco T (1999). Microanalysis of cardiolipin in small biopsies including skeletal muscle from patients with mitochondrial disease.. J Lipid Res.

[30] Morikawa T, Yasuno R, Wada H (2001). Do mammalian cells synthesize lipoic acid? Identification of a mouse cDNA encoding a lipoic acid synthase located in mitochondria.. FEBS Lett.

[31] Yi X, Maeda N (2005). Endogenous production of lipoic acid is essential for mouse development.. Mol Cell Biol.

[32] Fukuda T, Mishina Y, Walker MP, DiAugustine RP (2005). Conditional transgenic system for mouse aurora a kinase: degradation by the ubiquitin proteasome pathway controls the level of the transgenic protein.. Mol Cell Biol.

[33] Liang TJ, Reid AE, Xavier R, Cardiff RD, Wang TC (1996). Transgenic expression of tpr-met oncogene leads to development of mammary hyperplasia and tumors.. J Clin Invest.

[34] Wencker D, Chandra M, Nguyen K, Miao W, Garantziotis S (2003). A mechanistic role for cardiac myocyte apoptosis in heart failure.. J Clin Invest.

[35] Borutaite V, Brown GC (2003). Mitochondria in apoptosis of ischemic heart.. FEBS Lett.

[36] Chen ZJ, Pudas R, Sharma S, Smart OS, Juffer AH (2008). Structural enzymological studies of 2-enoyl thioester reductase of the human mitochondrial FAS II pathway: New insights into its substrate recognition properties.. J of Mol Biol.

[37] Williamson JR, Corkey BE (1979). Assay of citric acid cycle intermediates and related compounds–update with tissue metabolite levels and intracellular distribution.. Methods Enzymol.

[38] Gawehn K, Bergmeyer HU (1970). Anorganisches Phosphat. UV-spektrophotometrische Methode.. Methoden der enzymatischen Analyse.

[39] Lamprecht W, Trautschold I, Bergmeyer HU (1970). Adenosin-5′-triphosphat.. Methoden der enzymatischen Analyse.

[40] Massett MP, Berk BC (2005). Strain-dependent differences in responses to exercise training in inbred and hybrid mice.. Am J Physiol Regul Integr Comp Physiol..

