# Supplementary material for: Myocardial Overexpression of Mecr, a Gene of Mitochondrial FAS II Leads to Cardiac Dysfunction in Mouse
Source: PLoS One. 2009 May 18;4(5):e5589. doi: 10.1371/journal.pone.0005589 (PMC2680037; doi:10.1371/journal.pone.0005589)
Supplement: Table S1 — The rates of state 3 and 4 respiration of liver and heart mitochondria with various substrates. The liver and heart mitochondria were isolated as described [1] after 12 h fast. The incubations were carried out with 0.5–1.2 mg/ml of mitochondrial protein at 30°C in the appropriate medium (Materials and Methods) and 2 mM glutamate, 2 mM malate, 2 mM pyruvate, or 2 mM succinate. ADP was used at 1 mM concentration. The rate of oxygen consumption (nmol O2/min per mg protein) was monitored with Clark type oxgen electrode. The results are expressed as mean±standard deviation. The statistical significance was estimated using the Student's t test and the P-values are given. (0.02 MB DOC) [file pone.0005589.s001.doc]

**Supporting Information**

| **Liver** | **ADP -** | | **n=5** | **ADP +** | | **n=5** |
| --- | --- | --- | --- | --- | --- | --- |
|  | **wt** | **tg** |  | **wt** | **tg** |  |
| **malate + pyruvate** | 5.4 ± 0.3 | 5.4 ± 1.5 | P=1.00 | 12.1 ± 1.9 | 10.3 ± 2.8 | P=0.36 |
| **succinate** | 19.7 ± 2.9 | 19.5 ± 3.5 | P=0.89 | 63.5 ± 22.1 | 51.1 ± 11.6 | P=0.28 |
| **mal ate+ glutamate** | 9.5 ± 2.0 | 8.9 ± 2.2 | P=0.25 | 50.4 ± 8.2 | 41.2 ± 7.7 | P=0.08 |
| **Heart** | **ADP -** | | **n=5** | **ADP +** | | **n=5** |
|  | **wt** | **tg** |  | **wt** | **tg** |  |
| **malate + pyruvate** | 19.7 ± 2.7 | 17.1 ± 2.7 | P=0.28 | 82.9 ± 8.8 | 69.8 ± 30.9 | P=0.45 |
| **succinate** | 20.5 ± 7.9 | 18.2 ± 4.1 | P=0.56 | 10.8 ± 2.9 | 12.4 ± 1.5 | P=0.34 |
| **mal ate+ glutamate** | 18.0 ± 5.0 | 17.7 ± 5.0 | P=0.80 | 41.4 ± 11.7 | 36.8 ± 17.8 | P=0.49 |

**Table S1** The rates of state 3 and 4 respiration of liver and heart mitochondria with various substrates. The liver and heart mitochondria were isolated as described [1] after 12 h fast. The incubations were carried out with 0.5-1.2 mg /ml of mitochondrial protein at 30 ºC in the appropriate medium (Materials and Methods) and 2 mM glutamate, 2 mM malate, 2 mM pyruvate, or 2 mM succinate. ADP was used at 1 mM concentration. The rate of oxygen consumption (nmol O_2_ / min per mg protein) was monitored with Clark type oxgen electrode. The results are expressed as mean ± standard deviation. The statistical significance was estimated using the Student’s *t* test and the P-values are given.

1. [Pallotti F](http://www.ncbi.nlm.nih.gov/sites/entrez?Db=pubmed&Cmd=Search&Term=%22Pallotti%20F%22%5BAuthor%5D&itool=EntrezSystem2.PEntrez.Pubmed.Pubmed_ResultsPanel.Pubmed_DiscoveryPanel.Pubmed_RVAbstractPlus), [Lenaz G](http://www.ncbi.nlm.nih.gov/sites/entrez?Db=pubmed&Cmd=Search&Term=%22Lenaz%20G%22%5BAuthor%5D&itool=EntrezSystem2.PEntrez.Pubmed.Pubmed_ResultsPanel.Pubmed_DiscoveryPanel.Pubmed_RVAbstractPlus) (2001) Isolation and subfractionation of mitochondria from animal cells and tissue culture lines. [Methods Cell Biol](javascript:AL_get(this,%20'jour',%20'Methods%20Cell%20Biol.');) 65:1-35.
